# Putting the individual into reliability: Bayesian testing of homogeneous within-person variance in hierarchical models

**DOI:** 10.3758/s13428-021-01646-x

**Published:** 2021-11-23

**Authors:** Donald R. Williams, Stephen R. Martin, Philippe Rast

**Affiliations:** University of California, Davis, CA USA

**Keywords:** Bayesian, Varying reliability, Within-person variance, Bayes factor

## Abstract

Measurement reliability is a fundamental concept in psychology. It is traditionally considered a stable property of a questionnaire, measurement device, or experimental task. Although intraclass correlation coefficients (ICC) are often used to assess reliability in repeated measure designs, their descriptive nature depends upon the assumption of a common within-person variance. This work focuses on the presumption that each individual is adequately described by the average within-person variance in hierarchical models. And thus whether reliability generalizes to the individual level, which leads directly into the notion of individually varying ICCs. In particular, we introduce a novel approach, using the Bayes factor, wherein a researcher can directly test for homogeneous within-person variance in hierarchical models. Additionally, we introduce a membership model that allows for classifying which (and how many) individuals belong to the common variance model. The utility of our methodology is demonstrated on cognitive inhibition tasks. We find that heterogeneous within-person variance is a defining feature of these tasks, and in one case, the ratio between the largest to smallest within-person variance exceeded 20. This translates into a tenfold difference in person-specific reliability! We also find that few individuals belong to the common variance model, and thus traditional reliability indices are potentially masking important individual variation. We discuss the implications of our findings and possible future directions. The methods are implemented in the R package **vICC**

The particular problem studied here is the familiar analysis between and within groups....The assumption of a common [within-group] variance is usually made for convenience, rather than because it necessarily occurs in practice.— (pp. 1-11, Lindley, 1970)

## Introduction

Measurement reliability is an important aspect of repeated measurement designs, which are used extensively in the social-behavioral sciences. Their use spans from longitudinal studies that track individuals over their life span, to laboratory settings that can include hundreds of experimental trials for each person. Given that data are repeatedly obtained from the same individuals, they tend to result in non-independent structures, as measurements from the same individual are assumed to be more similar to one another than measurements from different individuals. This is commonly referred to as clustered data, in that units of observations are typically related to one another. These hierarchically structured data naturally lend themselves to assessing reliability by examining the degree of cluster cohesion.

Intraclass correlation coefficients (ICC) are commonly used to assess the level of agreement, or internal consistency, of observations organized into the same cluster (Bartko, 1966; McGraw & Wong, 1996). In repeated measurement designs, individuals are considered to be the cluster and the repeated measurements are nested within that cluster or person. In clustered data, the ICC serves as a reliability index as it quantifies the similarity of the data points *within*, relative to the difference *between* clusters (Bliese, 2000). As such, an ICC can characterize test-retest and inter-rater reliability (Shrout & Fleiss, 1979; Weir, 2005). It also corresponds to the proportion of total variance accounted for by the clustering (Musca et al., 2011). Another classical example comes from educational settings, where hierarchical data are often gathered from students that are nested within different schools (Morris, 2008; Theobald, 2018). In this case, the ICC would index the degree of similarity among students that attend the same school. This logic also extends to experimental designs, such as classic laboratory settings (Li, Zeng, Lin, Cazzell, & Liu, 2015; Pleil, Wallace, Stiegel, & Funk, 2018). A reliable experimental manipulation should induce similar responses from the same individual (Rouder, Kumar, & Haaf, 2019).

In order to compute the ICC, the different sources of variability need to be decomposed into within- and between-cluster variability (Hedges, Hedberg, & Kuyper, 2012). This can be accomplished either within an ANOVA framework (Shieh, 2012), or relatedly, within an unconditional hierarchical mixed-effects model with only random intercepts (i.e., “multilevel” models; Snijders & Bosker, 1993). In this current work we focus on the latter, because we will extend the classic mixed-effect model to allow the error terms to vary across and within clusters. This marks a drastic departure from the classical ICC literature that considers reliability to be *fixed* and *non-varying*. We present novel Bayesian methodology that allows for *testing* of varying intraclass correlation coefficients at the individual level. The foundation for this methodology is based upon the central idea of capturing individual differences with mixed-effects models.

Consider the case of a random intercepts only model. There are two sources of variation, that is,

1 $$ \begin{array}{@{}rcl@{}} \rho = \frac{{\sigma_{0}^{2}}}{{\sigma_{0}^{2}} + \sigma_{\epsilon}^{2}}. \end{array} $$

This is commonly referred to as ICC(1), and it can also be viewed as a reliability index for single scores that ranges from 0 − 1 (Shieh, 2016). Note that there are several ICC indices (Bartko, 1976) and each allows for asking specific questions about reliability. In this case, because the focus is on individual variation, we only consider ICC(1). We describe straightforward extensions in the discussion section (e.g., average score reliability). In Eq. 1, ${\sigma _{0}^{2}}$ is the between-person variance and $\sigma _{\epsilon }^{2}$ is the within-person variance, respectively. The latter is often referred to as measurement error. In cognitive inhibition tasks, for example, it captures trial-to-trial “noise” in reaction times. Thus, assuming that ${\sigma _{0}^{2}}$ is held constant, increasing $\sigma _{\epsilon }^{2}$ will necessarily decrease reliability (Hedge, Powell, & Sumner, 2018). This definition of ICC does not allow for the possibility of individual differences in reliability. However, if $\sigma _{\epsilon }^{2}$ is allowed to vary between individuals, this immediately leads to Eq. 1 representing the *average* reliability. Said another way, $\sigma _{\epsilon }^{2}$ can be viewed as the average within-person variance which suggests that it might not generalize to each person.

In the tradition of individual differences research it seems reasonable that the reliability of, say, an educational test or experimental manipulation, would not be the same for all people or all situations. This notion of varying reliability is not new and can be traced back nearly 50 years to a (working) paper entitled, “A Note on Testing for Constant Reliability in Repeated Measurement Studies”:

This paper discusses the potential usefulness of applying tests for the equality of variances (and covariances) to data from repeated measurement studies prior to estimating reliability components and coefficients ... Prior to actually applying some method of reliability estimation to a body of data from a repeated measurement study, consideration needs to be given to what assumptions are tenable concerning the stability of true and error variances (p.1; Silk, 1978).

To the best of our knowledge, this perspective has largely gone unnoticed in the literature. For example, an excellent paper by Koo and Li (2016) provides guidelines for selecting and reporting ICCs but it did not mention the implication of “Mean Squared Within” in an ANOVA framework, which is equivalent to ${\sigma _{1}^{2}}$ in Eq. 1. Of course, the ICC is often used descriptively (e.g., Noonan, Fairclough, Knowles, & Boddy, 2017) and assumptions are more important for significance tests (Bartlett & Frost, 2008). However, if there are notable deviations from the average, we argue that the estimate of reliability should account for this variation. This notion has serious implications for social-behavioral scientists: it provides the opportunity for researchers to *fully* characterize their measures with a fine-tooth comb.

For example, a researcher could use the presented methodology to extract certain people or simply quantify how many individuals the traditional ICC is representative of. Additionally, this could show that sometimes heterogeneity in within-person variance is so large, that a researcher may want to explore why that is the case. This work provides a tool—and the insight that in common situations there could be large individual differences in reliability. And now research psychologists can test this possibility.

To illustrate the importance of accounting for individual differences in ICCs, we will focus on cognitive inhibition tasks, where they are routinely computed to characterize reliability (Soveri et al., 2018; Strauss, Allen, Jorgensen, & Cramer, 2005; Wöstmann et al., 2013) and to justify subsequent statistical analysis steps (Hedge et al., 2018; Rouder et al., 2019). This literature serves as an excellent testing ground, although the presented methodology can be used for all hierarchically structured or clustered data. A recent debate surrounding the study of individual differences (Gärtner & Strobel, 2019; Hedge et al., 2018; Rouder et al., 2019), and in particular its relation to reliability formed the impetus for this current work. The emerging consensus is that reliability is too low (i.e., “noisy” measures) to adequately study individual variation in executive functioning.

However, the discussion has revolved almost exclusively around the mean structure and avoided the within-person variance structure altogether (i.e., $\sigma _{\epsilon }^{2}$). While the former reflects average reaction times, the latter refers to reaction time (in)stability—that is, consistency of executive functions. Indeed, Williams, Rouder, and Rast (2019) recently demonstrated that there were large individual differences in *consistently* inhibiting irrelevant information (Figure 3 in Williams, Rouder, & Rast, 2019). Although reliability was not considered in that work, those findings imply that there could be individual differences in reliability. This would present a quagmire. On the one hand, low reliability is thought to hinder our ability to study individual differences. But on the other hand, individual differences in reliability at the level of within-person variance may be a target for an explanatory model itself.

There is an interesting and storied literature on modeling within-person variance in hierarchical models (see references in: Cleveland, Denby, & Liu, 2003). The central idea goes back almost a century—that is, “[The quotidian variation] index may be of significance...since under the same test conditions individuals differ greatly in the degree of instability of behavior...” (p. 246; Woodrow, 1932). In other words, there are likely individual differences in within-person variability—which implies there is individual variation in reliability. These ideas are prominent in research areas that gather intensive longitudinal data (Hamaker, Asparouhov, Brose, Schmiedek, & Muthén, 2018; Hedeker, Mermelstein, & Demirtas, 2012; Rast & Ferrer, 2018; Watts, Walters, Hoffman, & Templin, 2016; Williams, Liu, Martin, & Rast, 2019). Indeed, to our knowledge, the notion of varying ICCs was first described in the context of ecological momentary assessment. In particular, Hedeker, Mermelstein, and Demirtas (2008) briefly described how the variances (e.g., ${\sigma _{0}^{2}}$ and ${\sigma _{1}^{2}}$) could be a function of covariates. This provided the foundation for Brunton-Smith, Sturgis, and Leckie (2017). That work in particular estimated group specific ICCs for interviewers using a hierarchical model (see Figures 2 and 3 in: Brunton-Smith et al., 2017).

There are several novel aspects of the present work. We propose a novel testing strategy that is based upon Bayesian model selection. This extends the approach of Brunton-Smith et al., (2017), where it was not possible to gain evidence for the null hypothesis. In our formulation, the null hypothesis can be understood as the common ICC model given in Eq. 1, but tested at the level of the within-person variance. In practical applications, this would allow a researcher to determine whether their estimate of reliability generalizes to each person. Further, another major contribution of this work is providing methodology to classify individuals into a common variance model. The importance of this cannot be understated. That is, we not only introduce methods for characterizing individual differences in reliability and rigorously testing for invariant reliability, but we also provide a model comparison strategy for assessing which (and how many) individuals belong to the ICC in Eq. 1. These are novel contributions. These methods also have serious implications for how we view past estimates of reliability. Namely, if a small proportion of individuals belong to the common ICC model, this would suggest that we have been masking important individual differences in reliability. We have also implemented the methods in the R package vICC.^1^

This work is organized as follows. In the first section we provide a motivating example. Our intention here is to demonstrate the *need* for varying ICCs, in addition to describing key aspects of the proposed model. This serves as the foundation for the remainder of the paper. We then introduce two models. The first tests for invariant within-person variance, whereas the second tests which (and how many) individuals belong to the common variance model. We then employ the proposed methodology in a series of illustrative examples. We conclude by discussing future directions for psychological applications.

## Motivating example

The presented methodology is based upon a straightforward extension to the traditional mixed-effects approach, which allows for partitioning the unexplained variance, or within-person variance, and allowing for the possibility of individual variation. The technique to do so is termed mixed-effects *location scale* model (MELSM, pronounced mel⋅zem; Hedeker et al., 2008, 2012). The location refers to the mean structure (e.g., response time) and the scale refers to the (within-person) variance. The MELSM *simultaneously* estimates sub-models to both structures (Rast and Ferrer, 2018; Williams & Rast, 2018). In this work, we build upon this foundation and introduce a spike and slab approach for both the random effects variance and the individual random effects for the within-person variance. To our knowledge, the spike and slab formulation has never been used for the variance structure. As we show below, this opens the door for answering novel research questions about the interplay between reliability and within-person variability in psychology.

First we present a relatively simple example with the goal of clarifying the central idea behind this work. We start with the customary ICC(1) model for single scores (1), and then proceed to extend the formulation to accommodate individual differences in within-person variability.

### Illustrative data

For the following we use data from a classical inhibition task that investigates the so-called “Stroop effect”. These data were first reported in von Bastian, Souza, and Gade (2016). They consist of 121 participants, each of which completed approximately 90 trials in total. About half of the trials were in the congruent condition, wherein the number of characters matched the displayed numbers–e.g., 22. The remaining trials were in the incongruent condition–e.g., 222. The outcome is reaction time for correctly identifying the number of characters.

### Mixed-effects model

For the *i* th person and *j* th trial, the one-way random effects model is defined as

2 $$ \begin{array}{@{}rcl@{}} y_{ij} = \beta_{0} + u_{0i} + \epsilon_{ij}, \end{array} $$

where *β*_0_ is the fixed effect and *u*_0*i*_ the individual deviation. More specifically, *β*_0_ is the average of the individual means and for, say, the first subject (*i* = 1), their respective mean response time is *β*_0_ + *u*_01_. The variance components are then assumed to follow

3 $$ \begin{array}{@{}rcl@{}} u_{0i} &\sim \mathcal{N}(0, {\sigma^{2}_{0}}) \\ \epsilon_{ij} &\sim \mathcal{N}(0, \sigma^{2}_{\epsilon}). \end{array} $$

Here the between-person variance ${\sigma ^{2}_{0}}$ captures the variability in the random effects *var*(*u*_0*i*_), and the individual deviations from the grand mean are assumed to be normally distributed with a mean of zero. Further, the residuals are also assumed to be normally distributed with a mean of zero and variance $\sigma ^{2}_{\epsilon }$. This readily allows for computing the ICC defined in Eq. 1 as $\frac {{\sigma _{0}^{2}}}{({\sigma _{0}^{2}} + \sigma _{\epsilon }^{2})}$.

### Mixed-effects location scale model

An implicit assumption of the standard mixed-effects model (e.g., Eq. 2) is that the residual variance is equal for each individual or group. Conceptually, this can be thought of as fitting *i* separate intercept only models, where each provides the respective reaction time mean, but then constraining the residual variance to be the same for each model.

The MELSM relaxes this assumption, in that each person is permitted to have their own mean and variance, that is,

4 $$ \begin{array}{@{}rcl@{}} y_{ij} &= \beta_{0} + u_{0i} + \epsilon_{ij} \\ \sigma_{\epsilon_{ij}}^{2} &= \exp[\eta_{0} + u_{1i}] \\ u_{1i} &\sim \mathcal{N}(0, {\sigma^{2}_{1}}) \end{array} $$

As indicated by the subscripts *i* and *j*, the error variance $\sigma _{\epsilon _{ij}}^{2}$ is now allowed to vary across *i* individuals and *j* trials given a log-linear model. The parameters in the scale model (the model for the error variance) are analogous to those in Eq. 2. *η*_0_ represents the intercept and defines the average of the individual variances (i.e., $\sigma _{\epsilon }^{2}$ in Eq. 3) and *u*_1*i*_ represent the random effect, that is, the individual departures from the fixed group effect. Again for the first subject (*i* = 1), *η*_0_ + *u*_11_ is the variability of their respective response time distribution. Note the exponent is used to ensure that the variance is restricted to positive values, and thus, is lognormally distributed (Hedeker et al., 2008).

It is also customary to assume that the random effects are drawn from the same multivariate normal distribution such that

5 $$ \begin{array}{@{}rcl@{}} \begin{bmatrix} u_{0i} \\ u_{1i} \\ \end{bmatrix} \sim \mathcal{N} \left( \begin{bmatrix} 0\\ 0 \\ \end{bmatrix}, \begin{bmatrix} {\sigma_{0}^{2}} & \rho\sigma_{0} \sigma_{1} \\ \rho\sigma_{0}\sigma_{1} & {\sigma_{1}^{2}} \\ \end{bmatrix}\right). \end{array} $$

Here ${\sigma _{0}^{2}}$ is the random effects variance of location intercepts and ${\sigma _{1}^{2}}$ is the random effects variance of the scale intercepts. Further, location and scale random effects are allowed to correlate (i.e., *ρσ*_0_*σ*_1_), thereby providing the mean–variance relation (Rouder, Tuerlinckx, Speckman, Lu, & Gomez, 2008; Wagenmakers & Brown, 2007; Williams, Rouder, & Rast, 2019).

### Individually varying reliability

Modeling the variance structure leads to individually varying ICCs. This is accomplished with a straightforward extension to Eq. 1, that is,

6 $$  \rho_{i} = \frac{{\sigma_{0}^{2}}}{{\sigma_{0}^{2}} + \exp[\eta_{0} + u_{1i}]} $$

Note that the subscript *i* denotes the *i* th individual. For example, with *i* = 1, this formulation would provide the person-specific estimate of reliability for the first subject. Further, in Eq. 6, the covariance between two observations from the same individual remains unchanged from the customary definition of ICC(1). In other words, the only modification is that the correlation is now expressed as a function of the individual, within-person variance, estimates. Of course, if there is not much individual variability in the variance structure (i.e., ${\sigma _{1}^{2}}$ is small), this would result in Eqs. 1 and 6 producing similar estimates. This is because a mixed-effects model is a special case of the MELSM, but with an implicit fixed intercept only model fitted to the variance structure.

Additionally, due to the hierarchical formulation, these reliability estimates will not be equivalent to solving Eq. 6 with the empirical variances. Indeed, in this model, the parameters share information (i.e., partial pooling of information) which can lead to improved parameter estimates due to shrinkage towards the fixed effect average (Efron & Morris, 1977; Stein, 1956). This is a defining feature of hierarchical estimation, and also applies to location-scale models.

### Application

**Fig. 1:**
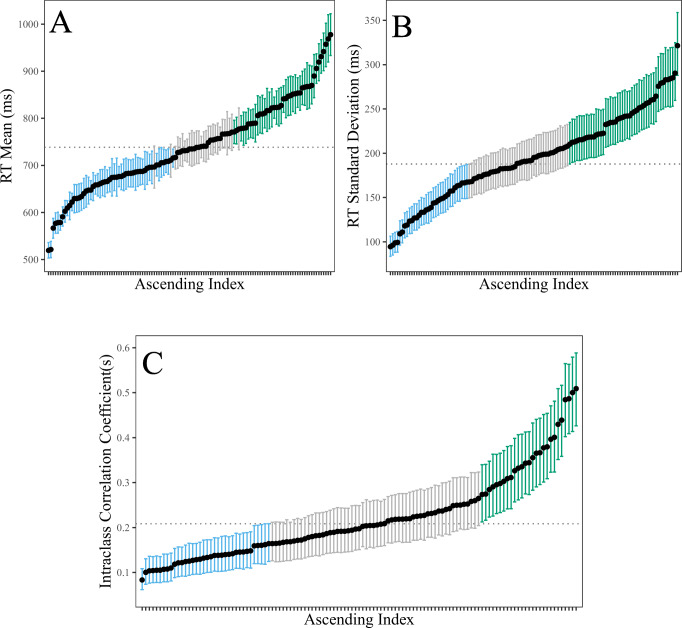
This plot motivates the *need* for individually varying ICCs. Panels **A** and **B** highlight individual variation in the reaction time means and standard deviations. The estimates are random intercepts for the location (mean) and scale (variance) sub-models, respectively. While the former are provided by a customary mixed-effects model (**A**), the variance structure is also assumed to be fixed and non-varying. In other words, that each person has the *same* reaction time standard deviation which corresponds to the dotted line in **B**. However, there are substantial individual differences in the scale model (**B**). This necessarily results in there being individual differences in reliability. This can be seen in panel **C**. The *dotted line* denotes the traditional ICC that assumes a common variance for each person. This masks important individual differences. In fact, there is a sixfold difference from the largest to the smallest ICC! The *bars* represent 90% CrIs for the hierarchical estimates. Those in either blue or green excluded the average

We fitted the MELSM and estimated varying ICCs with the R package vICC.^2^ The parameter estimates are displayed in Fig. 1. Panel A includes the individual means. The between-person variance (${\sigma _{0}^{2}}$) captures the variability in these estimates. Note that the slowest mean reaction time was 977 (ms) and the fastest was 519 (ms). As a point of reference, this is an 1.88-fold increase from the fastest to slowest individuals. These estimates can also be obtained from a standard mixed-effects model (2). Panel B includes the estimates of within-person variability. They are expressed on the standard deviation (SD) scale. In this case, the least consistent person had a SD of 321 (ms), whereas the most consistent had a SD of 94 (ms). This is a 3.41-fold increase from the least to most consistent individuals. Expressed as variance this is a 11-fold difference, which may be problematic, when considering the average (the dotted line) is used to compute reliability (1).

Panel C includes the varying intraclass correlation coefficients (defined in Eq. 6). Before describing these results, it is important to note that ICC(1) provides the lowest score among the different ICC definitions. We refer to Shieh (2016), where it was described how an ICC(1) = 0.20 could exceed 0.80 for average score reliability.^3^ The dotted line corresponds to the customary reliability estimate computed with the *average* within-person variance (ICC = 0.21, 90% CrI = [0.17, 0.25]). However, there were substantial individual differences in reliability. The smallest ICC was 0.08 and the largest was 0.51. In other words, for the classical Stroop task, there was a 6.10-fold increase from the least to most reliable individuals. This corresponds to over a 500% difference in reliability!

### Summary

This motivating example provides the foundation for the proceeding methodology. The central idea behind modeling individual varying variances was described, and in particular, how this relates to computing reliability from a one-way random effects model. The results demonstrated there were *substantial* individual differences in the within-person variance structure (panel B), which then necessarily results in individual differences in intraclass correlation coefficients or reliability. The degree of variation was not small, in that the 90% credible intervals excluded the average ICC for over half of the individuals (≈ 52*%*) in the sample. We argue this sufficiently motivates the *need* for investigating varying ICCs in psychological applications.

Importantly, the extent of this illustrative example parallels the work of Brunton-Smith et al., (2017). In particular, varying ICCs were computed for interviewers and then visualized in a similar manner as Fig. 1 (panel C). The rest of the paper includes our major and novel contributions. That is, we first describe methodology that tests for invariant within-person variance. This was not possible in Brunton-Smith et al., (2017), where the deviance information criteria (DIC) was used for model comparison (Spiegelhalter, Best, & Carlin, 2014). Our method allows for gaining (relative) evidence for the null hypothesis of invariant within-person variance with the Bayes factor. Further, for the goal of determining which (and how many) individuals belong to the common ICC model, we again focus on the within-person variance which directly targets the implicit assumption in Eq. 1. This is also based upon Bayesian hypothesis testing with the Bayes factor.

At this point, it is important to note that the decision on whether we have a common ICC model, as described in Eq. 1, or a varying ICC model, as described in Eq. 6, is obtained via the random effect *u*_1*i*_ in the within-subject variance model of Eq. 4. Another, seemingly intuitive approach, would be to use credible intervals computed from the person-specific ICCs (Fig. 1; panel C). However, this approach could only be used for detecting differences from the average ICC with an implicit null hypothesis significance test. Further, given that the varying ICC is a ratio of between-person and total variance, the posterior distribution also includes the uncertainty in the between-person variance. This can result in wider credible intervals. However, in our formulation, because the between-person variance is held *constant*, it follows that a difference in within-person variance results in a difference in reliability. The question at hand is therefore determined at the level of within-person variance and *before* reliability is computed.

## Bayesian hypothesis testing

Bayesian hypothesis testing is synonymous with model comparison. In contrast to classical testing (i.e., using *p*-values), the Bayesian approach provides a measure of *relative* evidence for which model is most supported by the data at hand. Thus, there must be at least two models under consideration–that is,

7 $$ \begin{array}{@{}rcl@{}} \underbrace{ \frac{Pr(\mathcal{M}_{a} | \mathbf{Y})}{Pr(\mathcal{M}_{b} |\mathbf{Y})}}_{\text{posterior odds}} = \underbrace{\frac{Pr(\mathbf{Y} |\mathcal{M}_{a})}{Pr(\mathbf{Y} |\mathcal{M}_{b})}}_{\text{Bayes factor}} \times \underbrace{\frac{Pr(\mathcal{M}_{a})}{Pr(\mathcal{M}_{b})}}_{\text{prior odds}}. \end{array} $$

In this formulation there are two models, ${\mathscr{M}}_{a}$ and ${\mathscr{M}}_{b}$, that can be thought of as competing predictions. Note that the prediction task is not for unseen data, as in commonly used information criteria (Vehtari, Gelman, & Gabry, 2017), but instead for the observed data **Y** (Kass & Raftery, 1995). The Bayes factor is commonly referred to as an *updating factor* (Rouder, Haaf, & Vandekerckhove, 2018), because it is multiplied by our prior beliefs about the models (i.e., the ratio prior model probabilities). It is common practice to assume equal prior odds, $Pr({\mathscr{M}}_{a}) / Pr({\mathscr{M}}_{a}) = 1$, which results in the Bayes factor and the posterior odds being equal to one another.

Although this intuitive framework appears to provide a simple approach for comparing models, it turns out that computing the Bayes factor can be quite challenging. It requires computing the marginal likelihood or the normalizing constant. Numerous methods have been proposed to compute this integral, for example Laplace’s approximation (Ruli et al., 2016), bridge sampling (Gronau et al., 2017), and Chib’s MCMC approximation (Siddhartha, 1995). Further, it is common to use conjugate prior distributions that provide an analytic expression for Eq. 7. This approach is limited to particular classes of models (Rouder & Morey, 2012), which limits its usefulness for location scale models.

### Spike and slab prior distribution

We employ the spike and slab approach for model comparison (George & McCulloch, 1993; Mitchell & Beauchamp, 1988; O’Hara & Sillanpää, 2009). This approach formulates model comparison in terms of a two-component mixture: 1) a “spike” that is concentrated narrowly around zero and 2) a diffuse “slab” component surrounding zero. The former can be understood as the null model, ${\mathscr{M}}_{0}$, whereas the latter is the unrestricted model, ${\mathscr{M}}_{u}$. Note that we prefer thinking of an unrestricted model and not necessarily a hypothesis (e.g., ${\mathscr{H}}_{1}$). Thus, in our formulation, the unconstrained model can be thought of as “not ${\mathscr{M}}_{0}$”.

A central aspect of this approach is the addition of a binary indicator, which in essence allows for switching between the two mixture components (i.e., transdimensional MCMC; Heck, Overstall, Gronau, & Wagenmakers, 2018). The proportion of MCMC samples spent in each component can then be used to approximate the respective posterior model probabilities. We refer interested readers to Rouder et al., (2018), that includes an excellent introduction to the spike and slab methodology. Further, O’Hara and Sillanpää (2009) presents an in-depth overview of the various specifications. Our specific application is clarified below.

## Model formulation

These model formulations were inspired by Haaf and Rouder (2018) and, in particular, Wagner and Duller (2012). The former used a spike and slab approach to investigate cognitive inhibition in, for example, the “Stroop effect”. In this case, they asked “...the posterior probability that all individuals are in the spike relative to the prior probability that all individuals are in the spike”. This was specifically for the priming effect, and they did not consider the variance structure (the focus of this work). On the other hand, Wagner and Duller (2012) considered a spike and slab approach for logistic regression models with a random intercept. This work also focused on the mean structure, and we extend their formulation to model within-person variability.

### Testing the common variance model

The common variance model refers to the implicit assumption of Eq. 1. Namely, that each person has the same (or similar) within-person variance. However, if there are individual differences in within-person variability, then the estimate of reliability should accommodate individual variation (6). The adequacy of a common ICC model can be inferred by testing the random effects variance in Eq. 5 (i.e., ${\sigma _{1}^{2}}$). That is, if there is evidence for *zero* variance in the scale intercepts (the spike component), this implies that Eq. 1 adequately describes each individual.

The presented applications use reaction time data that includes several repeated measures for each person. Thus, for the *i* th person and *j* th trial, the likelihood for each data set is defined as

8 $$ \begin{array}{@{}rcl@{}} y_{ij} & \sim \mathcal{N}(\beta_{0i}, \exp[\eta_{0i}]). \end{array} $$

This includes a location *β*_0*i*_ and scale *η*_0*i*_ intercept for each person. We employ the non-centered parameterization for hierarchical models–i.e.,

9 $$ \begin{array}{@{}rcl@{}} \beta_{0i} &=& \beta_{0} + \tau^{\mu} \cdot z^{\mu}_{i} \\ z^{\mu}_{i} &\sim& \mathcal{N}(0,1) \\ \beta_{0} &\sim& \mathcal{N}(0, 1) \\ \tau^{\mu} &\sim& \mathcal{S}\boldsymbol{t}^{+}(\nu = 10, 0, 1). \end{array} $$

Here we are not modeling the intercepts directly, but instead inferring them from a *latent* variable $z_{i}^{\mu }$. In Eq. 9, *β*_0_ is the fixed effect or average reaction time across individuals and *τ*^*μ*^ is the random effects standard deviation. They are each assigned a weakly informative prior distribution, with $\mathcal {S}\boldsymbol {t}^{+}$ denoting a half Student-t distribution. We then model the scale random effects similarly, but with the addition of $\tau ^{\sigma }_{*}$ and the Cholesky decomposition in order to include the correlation among the location and scale random effects,

10 $$ \begin{array}{@{}rcl@{}} \eta_{0i} &=& \eta_{0} + \tau^{\sigma}_{*}\left( z^{\mu}_{i}\rho + z^{\sigma}_{i}\sqrt{1-\rho^{2}}\right) \\ z^{\sigma}_{i} &\sim& \mathcal{N}(0,1) \\ z_{f} &\sim& \mathcal{N}(0, 1) \\ \rho &=& F^{-1}(z_{f}) \end{array} $$

Here *η*_0_ is the fixed effect or average within-person variability. This value is used to compute fixed and non-varying reliability (1). *ρ* captures correlation between the random effects, which is the mean–variance relation. We then place a standard normal prior distribution on *ρ*. This is accomplished by taking the inverse of the Fisher Z transformation (i.e., *F*^− 1^). The key difference from Eq. 9 is the introduction of $\tau ^{\sigma }_{*}$, which is the random effects standard deviation of the scale intercepts. This is where the spike and slab prior distribution is introduced–i.e.,

11 $$ \begin{array}{@{}rcl@{}} \tau^{\sigma}_{*} &=& \delta \text{ } \cdot \text{ } \tau^{\sigma} \\ \delta &\sim& \text{Bernoulli}(\pi) \\ \tau^{\sigma} &\sim& \mathcal{S}\boldsymbol{t}^{+}(\nu = 10, 0, 1). \end{array} $$

In this case, $\tau ^{\sigma } \sim \mathcal {S}\boldsymbol {t}^{+}(\nu = 10, 0, 1)$ is the slab component that can be understood as the unrestricted model (${\mathscr{M}}_{u}$). This formulation defines a Dirac spike at zero (i.e., a point mass). It was first introduced in Kuo and Mallick (1998). The key insight is that, for each MCMC iteration, a 0 or 1 is drawn from the Bernoulli distribution with the prior probability of sampling a 1 denoted *π*. To keep the prior odds at 1, *π* can be set to 0.5. Hence, this effectively allows for switching between a fixed effect *τ*^*σ*^ = 0 (${\mathscr{M}}_{0}$, i.e., common variance) and the random-effects model *τ*^*σ*^ > 0 (${\mathscr{M}}_{u}$)–i.e.,

12 $$ \tau^{\sigma}_{*} = \begin{cases} 0, & \text{if }\delta = 0 , \\ \tau^{\sigma}, & \text{if }\delta = 1 \end{cases}. $$

The posterior model probabilities can then be computed as

13 $$ \begin{array}{@{}rcl@{}} Pr(\mathcal{M}_{u} | \textbf{Y}) = \frac{1}{S} \sum\limits_{s = 1}^{S} \delta_{s}, \end{array} $$

where *S* = {1,...,*s*} denotes the posterior samples. Consequently, this formulation provides the necessary information for computing the Bayes factor defined in Eq. 7. For example, in the case of equal prior odds,

14 $$ \begin{array}{@{}rcl@{}} BF_{0u} = \frac{1 - Pr(\mathcal{M}_{u} | \textbf{Y})}{Pr(\mathcal{M}_{u} | \textbf{Y})}, \end{array} $$

results in the Bayes factor in favor of the spike component or the null hypothesis. We emphasize that this provides *relative* evidence compared the chosen unrestricted model (the slab), and it will also be influenced by the prior inclusion probability. Importantly, this is essentially variance selection for the within-person variance. As discussed before, zero variance (*τ*^2(*σ*)^ = 0) is implied by the customary ICC given in Eq. 1. Thus, if there is evidence for ${\mathscr{M}}_{u}$, then varying ICCs should be computed with Eq. 6.

### The membership model

The above approach focuses exclusively on the random effects variance and asks whether there is evidence for a common within-person variance. This question necessarily implies, “is there evidence for a common ICC or reliability?” that can be computed with the traditional ICC formulation (1). If there is evidence for varying ICCs, an additional question we can ask, relates to classification problems, such as, “which (or how many) individuals belong to the common variance model?” We term this the membership model.

The spike and slab approach has been used for computing posterior probabilities of individual random effects. In particular, Frühwirth-Schnatter, Wagner, and Brown (see Table 7; 2012) employed the technique for random intercepts in logistic regression. This work exclusively focused on the mean structure. We extend the general idea and model specification to the variance structure. This is a novel contribution.

The model formulation is almost identical to that described above (“Testing the common variance model”). The one change is that the indicator is removed from *τ*^*σ*^ and applied to the random effects–i.e.,

15 $$ \begin{array}{@{}rcl@{}} \eta_{0i}^{*} &=& z^{\mu}_{i}\rho + z^{\sigma}_{i}\sqrt{1-\rho^{2}} \\ \eta_{0i} &=& \eta_{0} + \tau^{\sigma} \big(\eta_{0i}^{*} \text{ } \cdot \text{ } \delta_{i}\big) \\ z^{\sigma}_{i} &\sim& \mathcal{N}(0,1) \\ \delta_{i} &\sim& \text{Bernoulli}(\pi). \end{array} $$

That slab component, or ${\mathscr{M}}_{u}$, is now comprised of various aspects of this model. For example, the prior distributions for *ρ*, *τ*_*σ*_, and the latent variable $z_{i}^{\sigma }$. Importantly, in reference to Eq. 10, the key difference is that the random effects standard deviation, *τ*^*σ*^, is always included in the model and the target for selection is the random scale effects (i.e., $\eta _{0i}^{*}$ in Eq. 15). In this way, the inclusion probability for each individual can be computed, in that, when not included in the model, their estimate is equal to the grand mean.

**Fig. 2:**
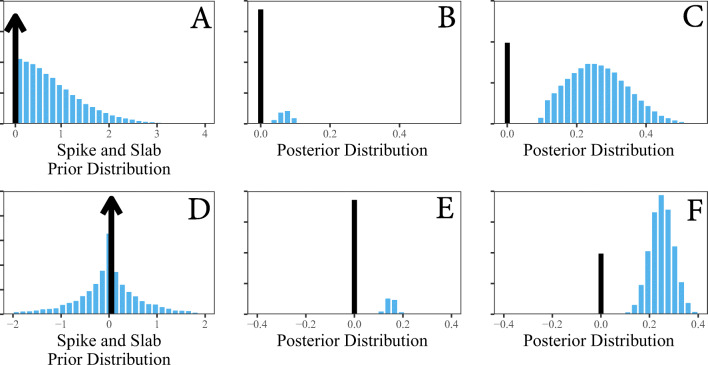
This plot clarifies the spike and slab approach for model comparison. Panels **A** and **D** include the prior distributions or competing models. The question at hand is then which most accurately predicts the observed data **Y**. The former (panel **A**) is for the random effects standard deviation *τ*^*σ*^. This provides a test for invariant within-person variance or non-varying random intercepts. The latter (panel **D**) is for the individual random effects *u*_1*i*_. This allows for testing whether each individual is equal to the average within-person variance, which is implied by traditional ICC formulations (1). In both panels, the *black line* represents the common variance model ${\mathscr{M}}_{0}$ (the spike component), whereas the unrestricted model ${\mathscr{M}}_{u}$ (“not ${\mathscr{M}}_{0}$”) is captured by the distributions. The spike and slab approach allows for “jumping” between the competing models. The number of posterior samples spent at each model can be used to approximate the respective model probabilities in Eq. 7. The remaining panels are hypothetical posterior distributions. For example, in panels **B** and **E**, 75% of the posterior samples were drawn from ${\mathscr{M}}_{0}$. This corresponds to $Pr({\mathscr{M}}_{0}|\textbf {Y}) = 0.75$. On the other hand, in panels **C** and **F**, 90% of the samples were drawn from ${\mathscr{M}}_{u}$. This corresponds to $Pr({\mathscr{M}}_{u}|\textbf {Y}) = 0.90$. These model probabilities can then be used to compute the Bayes factor with Eq. 7

To understand the implied prior distribution, and thus the unrestricted model, we sampled from the prior distributions. This is visualized in Fig. 2, where it was revealed that the slab component resembles a mixture between a normal and Student-t distribution. This results in a heavy-tailedness, which is often recommend for the slab component (e.g., Frühwirth-Schnatter et al., 2012; Wagner & Duller, 2012). The key aspects to focus on are the subscript to the indicator (*δ*_*i*_), which assigns each person a prior inclusion probability, and also the second line of Eq. 15. Recall that *δ*_*i*_ will either be 0 or 1. Thus, when a 0 is sampled, the portion after the fixed effect, or the average within-person variance (*η*_0_), drops out of the equation. In other words, for that particular MCMC sample, their estimate will then be equivalent to the average (*η*_0*i*_ = *η*_0_)–i.e.,

16 $$  \eta_{0i} = \begin{cases} \eta_{0}, & \text{if }\delta_{i} = 0 , \\ \eta_{0} + \tau^{\sigma}\left( z^{\mu}_{i}\rho + z^{\sigma}_{i}\sqrt{1-\rho^{2}}\right), & \text{if }\delta_{i} = 1 \end{cases}. $$

Importantly, since the average within-person variance is used to compute traditional ICCs, it follows that individual *i* is a member of the common ICC model (1) when *δ*_*i*_ = 0. Thus, for each iteration, this specification allows each individual to have their own person-specific estimate or the fixed effect average. Hence, each individual has a posterior probability of membership for belonging to the common variance model. Assuming equal prior odds, for example, this can then be used to compute the corresponding Bayes factor–i.e.,

17 $$ \begin{array}{@{}rcl@{}} BF_{0ui} = \frac{Pr(\eta_{0i} = \eta_{0}| \textbf{Y} )}{1 - Pr(\eta_{0i} = \eta_{0}| \textbf{Y} )}. \end{array} $$

We again emphasize that *η*_0_ corresponds to ${\sigma _{1}^{2}}$ in Eq. 1–i.e., $ \frac {{\sigma _{0}^{2}}}{({\sigma _{0}^{2}} + {\sigma _{1}^{2}})}$. Consequently, as we have argued, this implies membership to the common ICC model.

### Hypothetical example

This section clarifies our spike and slab implementation. First, it is important to note that there are a variety of possible specifications (O’Hara & Sillanpää, 2009). To our knowledge, only a point mass at zero has been used in psychological applications (Haaf & Rouder, 2018; Lu, Chow, & Loken, 2016, Rouder et al., 2018). However, it is possible to consider a mixture of continuous distributions (Carlin & Chib, 1995; Dellaportas et al., 2000), or described more recently, a hyperparameter formulation for the variances (Ishwaran & Rao, 2005, 2003). A simulation study comparing the alternative approaches can be found in Malsiner-Walli and Wagner (2011). For our purposes, we chose the Dirac spike approach for theoretical reasons (exactly zero) and also in reference to the summary provided in O’Hara & Sillanpää (see Table 1: 2009). Namely, the Dirac spike was comparable in terms of computational feasibility and performance, while also providing estimates of exactly zero.

For illustrative purposes, we plotted competing models in Fig. 2. Panel A includes ${\mathscr{M}}_{0}$ and ${\mathscr{M}}_{u}$ that were described above (“Testing the common variance model”). In particular, these competing models test whether there is a common within-person variance. This is implied when computing ICC(1) (i.e., Eq. 1). The black line represents the spike component (${\mathscr{M}}_{0}$), whereas the blue distribution is the slab component (${\mathscr{M}}_{u}$). Panel B includes a hypothetical posterior distribution. In this case, after conditioning on the observed data **Y**, there would be evidence for the spike $Pr({\mathscr{M}}_{0} | \textbf {Y}) = 0.75$. Assuming equal prior odds, this corresponds to evidence in favor of the null hypothesis of a common within-person variance (*BF*_0*u*_ = 3), which implies that there is (relative) evidence for a common ICC that is captured by the average within-person variance. This inference follows the customary guidelines provided in Kass and Raftery (1995) and Jeffreys (1961). On the other hand, panel C includes an example posterior that would provide evidence for vary within-person variance. Namely, the posterior model probability for the slab component is $Pr({\mathscr{M}}_{u}| \textbf {Y}) = 0.90$, which corresponds to *BF*_*u*0_ = 9.0 (assuming equal prior odds).^4^ Thus, in this hypothetical example, there is evidence for individual differences in within-person variance, and as a result, there is also evidence in favor of computing varying ICCs.

This notion also applies to the individual random effects, or the membership model, but in this case the spike component corresponds to the fixed effect average. This is plotted in Fig. 2 (panels C, D). To avoid redundancy it is further summarized in the caption.

## Illustrative examples

We now apply the proposed methodology to two classical inhibitions tasks. The data are different from above (“Motivating example”). In particular, there are fewer people (*n* = 47) but (substantially) more repeated measurements from the same individual. They were originally collected and used in Hedge et al., (2018), and they were also analyzed in Rouder et al., (2019). Both of these papers raised concerns about the study of individual differences in relation to measurement reliability. They also focused on the mean structure. We use the same data to characterize individual variability in the within-person variance structure, and thus, measurement reliability.

### Data set 1: flanker task

Rather than reword the study description, we instead directly quote the original study authors. The task protocol was succinctly described in Hedge et al., (2018):

Participants responded to the direction of a centrally presented arrow (left or right) using the ∖and / keys. On each trial, the central arrow (1 cm × 1 cm) was flanked above and below by two other symbols separated by 0.75 cm...Flanking stimuli were arrows pointing in the same direction as the central arrow (congruent condition), straight lines (neutral condition), or arrows pointing in the opposite direction to the central arrow (congruent condition). Stimuli were presented until a response was given (p. 1196).

We computed the reliability of correct responses for the congruent, incongruent, and neutral responses in separate models. We followed the protocol described in Haaf and Rouder (2017): reaction times less than 0.2 and greater than 2 s were removed from the data.

### Data set 2: Stroop task

Hedge et al., (2018) included several cognitive tasks that are thought to measure the same thing. We chose this task in particular because it most closely paralleled the flanker task. Thus we could fit models to the same types of responses. We again directly quote the experimental protocol from Hedge et al., (2018):

Participants responded to the color of a centrally presented word (Arial, font size 70), which could be red (z key), blue (x key), green (n key), or yellow (m key). The word could be the same as the font color (congruent condition), one of four non-color words (lot, ship, cross, advice) taken from Friedman and Miyake (2004) matched for length and frequency (neutral condition), or a color word corresponding to one of the other response options (incongruent). Stimuli were presented until a response was given. Participants completed 240 trials in each condition (720 in total) (p. 1196).

This task included the same number of trials for each condition as the flanker task (i.e., 240). We again analyzed only the correct responses for congruent, incongruent, and neutral responses. These data were also cleaned following Haaf and Rouder (2017).

### Software and estimation

The models were fitted with the R package vICC, which uses the Bayesian software JAGS (Plummer, 2016). Note that an advantage of JAGS is the ability to fit spike and slab models in particular (see the appendices in: Ntzoufras, 2002; O’Hara & Sillanpää, 2009). For each model, we obtained 20,000 samples from the posterior distribution, from which we discarded the initial burn-in period of 5000 samples. This number of samples provided a good quality of the parameter estimates and stable posterior model probabilities. We restrict our focus to the scale model and also the varying ICCs.

### The common variance model

Before describing these results, first recall that the central focus of this work is the within-person structure. The idea is that, because reliability in repeated measurement studies is computed with the *average* within-person variance (e.g., mean squared within), it is a natural target for “putting the individual into reliability”. That is, if there are large deviations from the average “error”, then person-specific, varying ICCs, can be employed to gain further insights into measurement reliability.

**Fig. 3:**
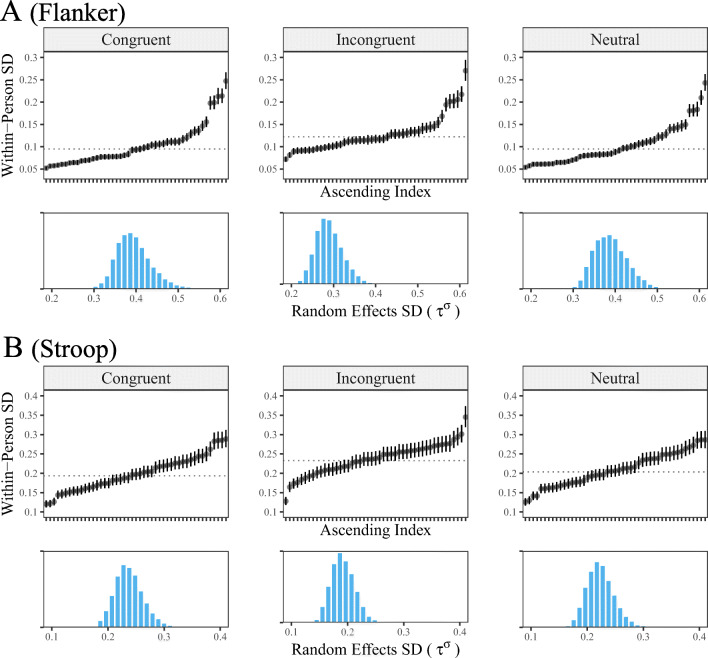
The points correspond to person-specific within-person variability that is expressed on the standard deviation scale. The *dotted lines* denote the average within-person SD and the bars are 90% CrIs. This reveals substantial individual differences in the scale model for both tasks and all three outcomes. This necessarily results in there being individual differences in reliability. Importantly, the traditional ICC assumes a common variance for each person that corresponds to the *dotted lines*. This masks important individual differences. The histograms are the posterior distributions of *τ*^*σ*^, which is the random effects SD for the scale model. It captures the spread in individual variability, in that, if *τ*^*σ*^ = 0, this suggests there is invariant within-person variance. For both tasks and all three outcomes, the posterior probability for the common variance model was zero, which results in an infinite Bayes factor in favor of varying within-person variance. This can be inferred from the histograms: The posterior distributions are well separated from zero

Figure 3 includes the individual, random effects, for the variance structure. Note that the estimates are reported as reaction time standard deviations, which eases interpretation. Importantly, the dotted line corresponds to the fixed-effect, or the average within-person variability. This estimate would traditionally be used to compute the ICC given in Eq. 1. This implicitly assumes that each person (or group) can be adequately described by the average. However, as revealed in Fig. 3, there are considerable individual differences in within-person variance. As an example, panel A includes the individual estimates for the congruent responses in the flanker task, where there is a fivefold difference from the least (0.05) to most variable individuals (0.25). There are recommendations pertaining to when unequal variances become problematic; for example, a common “rule of thumb” is when the ratio between the largest to smallest variance exceeds 3 or 4. In this case, when expressed on the variance scale, the maximum-minimum ratio exceeded 20!

Moreover, the individual, within-person variability estimates, revealed a similar pattern between all three outcomes and both tasks. Namely, there were notable individual differences in the variance structure. This suggests that the inherent variation is not a peculiarity of one data source, task, or response type. This insight was made possible with the presented methodology.

The histograms correspond to the random effects standard deviation for the scale intercepts (*τ*^*σ*^). This captures the spread of the within-person variances, that are assumed to be sampled from the same normal distribution. Further, *τ*^*σ*^ was subject to spike and slab model comparison. Here the spike component, or ${\mathscr{M}}_{0}$, corresponds to a fixed effect model (*τ*^*σ*^ = 0). This corresponds to the assumption of homogeneous within-person variance. On the other hand, the slab component, ${\mathscr{M}}_{u}$, corresponds to the unrestricted model that permits heterogeneity in the variance structure. Our intention was originally to compute the Bayes factor, given in Eq. 7, for the competing models. However, for each outcome and task, the probability of the slab component was 1.0. Thus the Bayes factors were all infinite! This can be seen in Fig. 3. The posterior distributions are well separated from zero, which indicates overwhelming (relative) evidence for heterogeneous within-person variances.

### The membership model

The membership model builds upon the common variance model. Namely, it allows for determining which (and how many) individuals are adequately described by the average within-person variance or mean squared within in an ANOVA framework. This is the implicit assumption of computing Eq. 1, in that this measure of reliability utilizes a common variance.

**Fig. 4:**
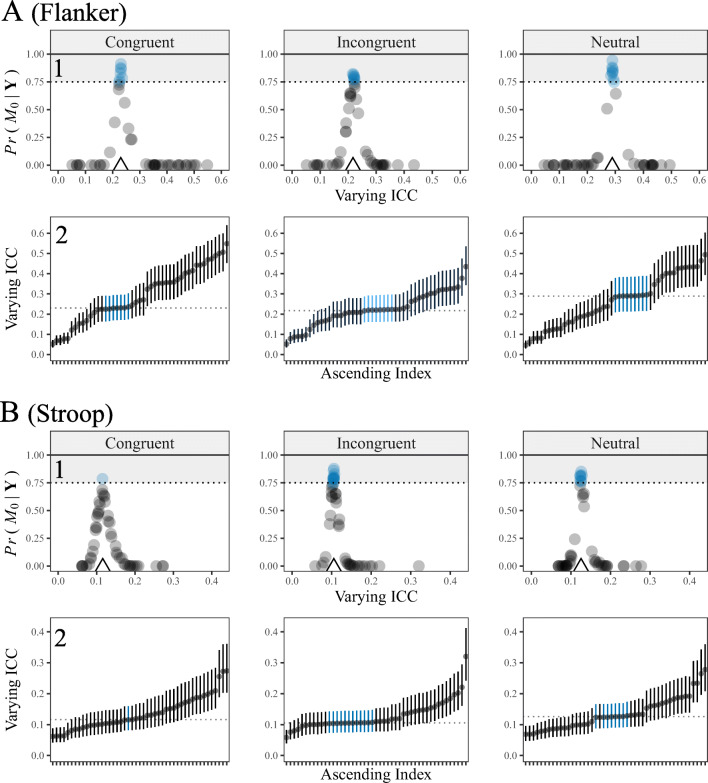
Results from the membership model. In row 1, the posterior probabilities in favor of the common variance model are on the *y*-axis and the varying ICCs are on the *x*-axis. The *shaded region* corresponds to a Bayes factor greater than three and the *triangle* denotes the average ICC. The accuracy of the model can be inferred from this plot. Namely, the posterior probabilities in favor of ${\mathscr{M}}_{0}$ gradually became smaller for larger deviations from the average ICC. Indeed, $Pr({\mathscr{M}}_{0} | \textbf {Y}) = 0$ corresponds to $Pr({\mathscr{M}}_{u} | \textbf {Y}) = 1$. In row 2, the points are person-specific ICCs, the *dotted lines* denote the average ICC, and the *bars* are 90% CrIs. The *blue bars* and*points* are individuals that belong to the common variance model. For demonstrative purposes, this was determined with a Bayes factor greater than three. This reveals that few people belong to the common variance model, which is used to compute Eq. 1, and that there are individual differences in reliability

Figure 4 includes these results. We focus on row 1. The varying ICCs can be seen on the *x*-axis, where the average ICC is denoted with a triangle. This shows the spread of measurement reliability in these data. For example, panel A includes congruent responses for the flanker task. Here the lowest ICC was 0.05 and the highest was 0.55. This corresponds to over a tenfold increase from the least to most reliable measurements for this outcome and task. Note that the other panels had less variability, but the maximum-minimum ratio always exceeded 3.

The *y*-axis includes the posterior probabilities in favor of belonging to the common variance model. That is, the evidence in the data for each person being accurately described by the average within-person variance. The shaded grey region corresponds to a Bayes factor of 3, which is a point of reference that indicates “positive” evidence for ${\mathscr{M}}_{0}$ (Kass & Raftery, 1995). It was revealed that very few people across all outcomes and both tasks belong to the common variance model, whereas roughly half were determined to belong to the slab component. Indeed, for many individuals, the posterior probability of the spike was zero. Said another way, the probability of belonging to the slab component was 1 (an infinite Bayes factor).

Figure 4 was conceptualized with a secondary goal of illustrating the central idea behind this model (again row 1). This can be seen by noting both axes in relation to the average ICCs that are denoted with triangles. For example, the highest posterior probabilities are centered directly above the average reliability. This is expected, in that, as we have highlighted throughout this work, the ICC is computed from the average within-person variance. Thus, for those that belong to the common variance model, their respective reliability will be very similar to the fixed and non-varying ICC given in Eq. 1. Further, the posterior probabilities in favor of ${\mathscr{M}}_{0}$ gradually became smaller for larger deviations from the average ICC. Said another way, for increasingly larger differences from the average ICC, the posterior probabilities also became larger for the slab component or the unrestricted model ${\mathscr{M}}_{u}$ (Fig. 2; panel D).

### Robustness check

Thus far, we have not discussed a decision rule for the spike and slab approach for model comparison. This is intentional, in that Bayesian inference is focused on the weight of evidence and is thus decoupled from making decisions (Morey, Romeijn, & Rouder, 2016). Further, the most common decision rule does not entail computing a Bayes factor, but instead the median probability model is perhaps the most popular choice (Lu et al., 2016; Mohammadi & Wit, 2015). Here, variables are selected with $Pr({\mathscr{M}}_{a} | \textbf {Y}) > 0.50$, although this was originally proposed for the goal of future prediction and it assumed an orthogonal design matrix (Barbieri & Berger, 2004). We refer to Piironen and Vehtari (2017), where violations of this assumption were investigated and compared to the most probable model (among other methods).

Regardless of the evidentiary threshold or decision rule, however, it will be influenced by the prior distribution to some degree. This is not a limitation, but instead, in our view, this can strengthen claims with counter-factual reasoning. In what follows, we adopt the perspective of trying to persuade a skeptic to the central implication of the results–i.e., relatively few people belong to the common variance model, which (perhaps) calls into question traditional reliability indices.

To convince her, we performed a sensitivity analysis to check the robustness of the results. In this work, she was primarily concerned with two sources that could influence the resulting inference. The first is the unconstrained model, ${\mathscr{M}}_{u}$, or the slab component. And the second is the prior inclusion probability *π*. To address these concerns, we varied the assumed prior distributions for the flanker task congruent responses. Recall that the prior distribution for the individual random effects is a scale mixture (Fig. 2; panel D). We thus increased the scale for the prior on *τ*^*σ*^, *ν* ∈{1,2 and 3}, which increasingly results in more diffuse priors. This could hinder “jumps” to the slab component (O’Hara & Sillanpää, 2009), and when assuming a ground truth, this is known to favor the null hypothesis of a common variance (Gu, Hoijtink, & Mulder, 2016). Furthermore, she had a strong belief in the adequacy of the common variance model. This was expressed as $Pr({\mathscr{M}}_{0}) = 0.80$, although we assumed a range of prior model probabilities. We used a decision based on the posterior odds exceeding 3.

**Fig. 5:**
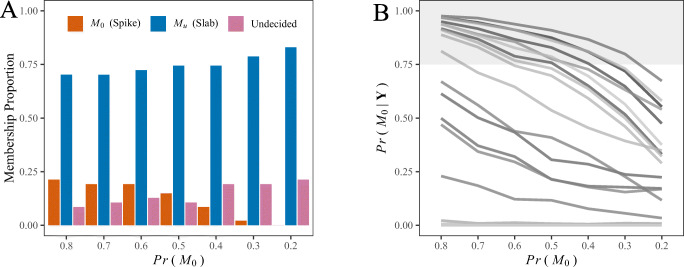
Results from the robustness check. In panel **A**, the prior model probabilities for the common variance model are on the *x*-axis and the membership proportion is on the *y*-axis. The latter refers to the proportion of individuals that belong to the competing models (i.e., ${\mathscr{M}}_{0}$ vs. ${\mathscr{M}}_{u}$). Recall that ${\mathscr{M}}_{0}$ is the common variance model (the spike) and ${\mathscr{M}}_{u}$ is the varying variance model (the slab). For demonstrative purposes, membership was determined with a posterior odds greater than three. This reveals that classifying individuals was robust to the prior distribution. Moreover, the key implication of this work was strengthened (i.e., that few people are adequately described by the average within-person variance), in that, even with $Pr({\mathscr{M}}_{0}) = 0.80$, the majority of individuals still belonged to ${\mathscr{M}}_{u}$. In panel **B**, the prior model probabilities for the common variance model are on the *x*-axis and the posterior model probabilities are on the *y*-axis. Each *line* is an individual (*n* = 47) and the *shaded region* corresponds to a posterior odds greater than three. Note that the majority of individuals are overlapping each other at a posterior probability of zero, which indicates essentially no support for the null model. For others, however, the posterior probabilities were sensitive to the prior probabilities. The former gradually decreased with smaller prior probabilities for ${\mathscr{M}}_{0}$. Importantly, only one person switched from being undecided and to the common variance model ${\mathscr{M}}_{0}$

Figure 5 includes the results. Note that the random effects standard deviation, *τ*^*σ*^, was robust to all prior specifications we considered, with each resulting in a posterior probability of 1 in favor of varying intercepts, or individual differences in the variance structure, for the scale model. Consequently, we restrict our focus to the membership model. Further, because there was essentially no difference between the various scale parameters we only discuss *ν* = 1. This was used in the primary analysis. Panel A shows the proportion of individuals that belong to each mixture component, as function of the prior probability for the common variance model. This reveals the classification results were consistent, for example even with $Pr({\mathscr{M}}_{0}) = 0.80$, the proportion of individuals belonging to ${\mathscr{M}}_{0}$ did not exceed 25%. And the majority of individuals belonged to ${\mathscr{M}}_{u}$, or the slab component, regardless of the prior odds. Panel B shows the posterior probabilities as a function of the prior probabilities. The shaded area corresponds to the critical region. In this case, the probabilities in favor of ${\mathscr{M}}_{0}$ gradually decreased to eventually there being zero individuals belonging to the spike component. Further note that, with $Pr({\mathscr{M}}_{0}) = 0.80$, that corresponds to a strong belief, only one person changed from undecided to the common variance model.

Together, this points towards robustness of the results that ultimately satisfied the skeptic. And this also highlights that our membership model works nicely for the goal at hand, in that the various models produced the expected results. For example, in panel A, the largest proportion of individuals belonging to ${\mathscr{M}}_{0}$ was observed with the highest prior probability. And the proportion gradually diminished with decreasing prior probabilities. A similar pattern was revealed in panel B. In practical applications, we recommend that in lieu of strong prior beliefs, or a prior distribution that adequately reflects a hypothesis, similar robustness checks can be performed. These are implemented in the R package vICC.

## Discussion

In this work, we proposed a novel testing strategy for homogeneous within-person variance in hierarchical models. The primary motivation for developing this methodology was for applications in measurement reliability. We argued that reliability in repeated measurements is often computed without considering the implicit assumption of a common within-person variance, which is typically assumed to be the case in ANOVA and hierarchical models, and thus also assumed in traditional formulations for computing intraclass correlation coefficients. Our method, for characterizing individual differences, specifically targeted reliability at the level of the within-person variance structure. This was accomplished by extending the traditional mixed-effects approach to include a sub-model that permits individual differences in within-person variance.

Moreover, Bayesian hypothesis testing, and in particular the spike and slab approach, was used for comparing competing models. On the one hand, our model comparison formulation posited a common (within-person) variance that is represented by a spike component. On the other hand, the unrestricted, or the varying within-person variance model, was represented by a slab component. This approach allows researchers to assess (relative) evidence for the null hypothesis of a common variance, which is assumed to be representative of each individual when computing traditional measures of reliability. Further, we also introduced the membership model. Here the goal was to explicitly determine which (and how many) individuals belong to the common variance model. The importance of these contributions cannot be understated. First, a researcher can determine the generalizability of measurement reliability in their repeated measurement studies. Second, individual differences in within-person variance provides a natural target for improving reliability. For example, by developing methodology to hone in the final sample to either exclude individuals determined to be unreliable or considering sub-groups that have a common variance.

### What is sufficient evidence?

The presented approach did not employ a hard and fast threshold for determining whether the null hypothesis should be “rejected”. For example, although we used the Bayes factor threshold of three as a reference point in Fig. 4, the overall message was that reliability varies which could be surmised from the posterior probabilities in relation to the individual level ICCs. In practice, however, it may be desirable to directly make a decision regarding which individuals share a common variance. To this end, there are two strategies. The first is to follow the guidelines provided in Kass and Raftery (p. 777, 1995), which are commonly used in psychology. Here a Bayes factor of three is considered “positive evidence” that will typically be more conservative than a significance level of 0.05. The second approach is to use a posterior probability greater than 0.50 (or a Bayes factor of one) that results in the “median probable model” (Barbieri & Berger, 2004). This approach can be used if a decision is *necessary*, given that using a Bayes factor of three can result in ambiguous evidence (neither hypothesis was supported).

Furthermore, in the membership model, there is the issue of multiple comparisons, given that potentially hundreds of tests are being conducted. In a Bayesian framework, this can be remedied by adjusting the prior probabilities which is straightforward in the spike and slab formulation (e.g., by making *π* in Eq. 11 smaller). We refer interested readers to Scott and Berger (2010), that provides a full treatment of multiplicity control, and note that our package **vICC** allows for seamlessly changing the prior inclusion probabilities.

### A note on sample size

Due to providing two models, it is worth discussing how the sample size would affect the posterior of each. For the test of a common variance, the random effects variance is the target, and thus it is ideal to have many individuals (or units). Intuitively, this is because the variance is being estimated from the random effects, which will be less accurate with few subjects. As a result, it will be harder to gather evidence for varying within-person variance, even when the null hypothesis is false. On the other hand, for the membership model, it is advantageous to have many observations from each person. This is because the target is the individual effects, such that more data from each subject will reduce uncertainty that then translates into more decisive evidence. Together, the target of selection should be considered when deciding how to gather observations. Our illustrative examples indicated that as few as 50 subjects can provide a clear picture of varying reliability, so long as there are many repeated measurements. Going forward, it would be informative to determine how few repeated measurements can be used to fit the proposed models. In our experience, data common to cognitive tasks in particular will be more than sufficient.

### Implications

The utility of our method was demonstrated on cognitive inhibition tasks. As we mentioned in the Introduction, this literature is an excellent testing ground for assessing individual differences in within-person variance. Namely, in Rouder et al., (2018) and Hedge et al., (2018), it was argued that reliability was not high enough to adequately study individual differences. However, reliability was considered a fixed and non-varying property of these same tasks. This work demonstrated that there are substantial individual differences in the variance structure, and that reliability can be the target of an explanatory model.

Further, we argue our findings present a challenge to the notion that individual differences studies in these tasks are necessarily “bound to fail” (Rouder et al., 2018). First, there are large individual differences in the variance structure. This has not been considered in this debate, which is unfortunate, because within-person variance could be a key aspect of executive functions such as inhibition. In certain tasks the “stability of instability” has been shown to have adequate, and in some cases, excellent retest reliability (Fleming, Steiborn, Langner, Scholz, & Westhoff, 2007; Saville et al., 2011). This points towards a possible disconnect between methodological and substantive inquires, in that, for the latter, *intra* individual variation (IIV) is often studied in these same tasks (Duchek, Balota, Tse, Holtzman, Fagan, & Goate, 2009; Fehr, Wiechert, & Erhard, 2014; Kane et al., 2016). Second, and more generally, if a researcher is interested in individual differences, they have to at least approach the individual level. This is not easily accomplished with a traditional mixed-effects model (p. 17 in: Hamaker, 2012). This has been an ongoing debate in longitudinal modeling in particular, but to our knowledge, it has not been considered in these recent debates in cognitive psychology. We refer interested readers to Molenaar (2004) and Hamaker (2012). Third, from our perspective, a satisfactory answer to the question of individual differences in, say, the “Stroop effect,” would require addressing the extreme heterogeneity in within-person variance (and thus reliability) that is apparently a defining feature of these tasks. 5 This work not only raised this question, but the presented methodology and the conceptual framework of varying reliability can serve as a guiding light for answering this important question.

### An alternative perspective

It would be remiss of us to not offer an alternative perspective. It is customary to view the residuals as mere “noise” and perhaps measurement “error”. For example, that trial-to-trial fluctuations are a nuisance to understanding the latent process. On the other hand, there is a large literature that views these same fluctuations as a key aspect of the construct. A good example is personality traits, that were customarily considered fixed, but now an active area of research revolves around within-person variability of these traits (i.e., the fluctuations; Fleeson, 2001; Hutteman, Back, Geukes, Küfner, & Nestler, 2016; Williams, Liu, Martin, & Rast, 2019). So rather than there being individual differences in reliability, the alternative perspective is to view these as individual differences in stability. That is, individuals with larger residual variance are relatively more volatile or inconsistent, which in of itself, is inferential. In fact, reaction time variability is often studied in substantive applications, for example, it is thought to be a core feature of the ADHD cognitive profile (Borella, De Ribaupierre, Cornoldi, & Chicherio, 2013; Tamm et al., 2012). This is diametrically opposed to classical test theory (CTT), and thus the reliability literature, where measurements are construed as a “true” score plus error. And note that “individual differences in IIV inherently violate core assumptions of CTT” (p. 3; Estabrook, Grimm, & Bowles, 2012). We think this offers a plausible alternative worth considering:

It is quite possible that we insist on unduly expensive measurement accuracy in some situations where we do not need it, because of limitations imposed by the intra-individual variation. At the same time, we may be blissfully unaware of the need for more refined measurement in certain other situations. (p. 159, Henry, 1959a)

### Limitations

The idea behind this work was to put the “individual into reliability”. This addresses recent calls in the social-behavioral sciences to place more emphasis at the individual level (Molenaar, 2004). In doing so, we assumed the same functional form for each person. However, completely separating group and individual dynamics is not easily achieved. In our experiences, we have found that the MELSM provides an adequate compromise between aggregation approaches and person-specific models. Further, our approach does not separate within-person variability from measurement error. This is not only an “issue” of this work, but it also applies to computing intraclass correlation coefficients more generally–i.e., “...variations between and within individuals characterize *behavior*, which may or may not be reliable regardless of measurement error” (Henry, 1959b). This hints at the notion of *random* vs. *systematic* error, which are not easily teased apart in mixed-effects models. One thought, assuming that a necessary ingredient of the latter is reproducibility (at minimum), is to compute a naive correlation between response types. We investigated this possibility in the flanker task, and found large correlations between not only the within-person variance but also the person-specific reliabilities. At the individual level, this suggest that there is some degree of systematicity.

### Future directions

The proposed methodology provides a foundation for further quantitative advances. First, it is important to note that we did not directly target reliability, but instead an aspect of reliability. This is by design. There is some literature on testing for differences in ICCs. One strategy is to simply compare Fisher *z*-transformed correlations (Konishi & Gupta, 1989). These approaches are typically for comparing groups such as countries (Mulder & Fox, 2019) or schools located in different areas (e.g., rural vs. urban; Hedges & Hedberg, 2007). On the other hand, we view our methodology as more foundational. Rather than take reliability as a fixed property, that is, our approach allows for an uncanny attention to detail by explicitly *modeling* the variance components. The MELSM allows for predicting both the between and within-person variance structures. Thus the present framework allows for probing reliability at the level of both the numerator and denominator of Eq. 1—i.e.,$\frac {{\sigma _{0}^{2}}}{({\sigma _{0}^{2}} + {\sigma _{1}^{2}})}$. Second, the testing strategy for within-person variance can seamlessly be extended to all forms of intraclass correlation coefficients. Thus our work provides the necessary ingredients for considering individual differences in reliability more generally. These ideas point towards our future work.

### Conclusions

Measurement reliability has traditionally been considered a stable property of a measurement device or task. This framework does not allow for the possibility of individual variation, because it assumes the residual variance is fixed and non-varying. We demonstrated that there can be large individual differences in within-person variance, which necessarily implies the same for reliability. Before computing reliability in hierarchical models, we recommend that researchers first assess whether a common variance is tenable. And if not, varying intraclass correlation coefficients should be computed to fully capture individual level variation in reliability.

## Footnotes

1 **v** arying **I** ntraclass **C** orrelation **C** oefficients

2 Note that we excluded the prior specification from this section for simplicity. The posterior distributions are summarized with means and 90% credible intervals. The prior distributions are described in the next section.

3 We investigated this with data provided in Hedge et al., (2018). They used average scores for each person across two sessions to compute retest reliability. Here we noted their reported estimates of around 0.70 translated to (roughly) 0.20 for the single scores.

4 $BF_{u0} = \frac {Pr({\mathscr{M}}_{u}| \textbf {Y})}{Pr({\mathscr{M}}_{0}| \textbf {Y})} = \frac {Pr({\mathscr{M}}_{u}| \textbf {Y})}{1 - Pr({\mathscr{M}}_{u}| \textbf {Y})}$

5 Note that Hedge et al., (2018) considered retest reliability of mean scores at two different occasions. We investigated the within-person variance structure, using the same analysis of Hedge et al., (2018), where we found a 74-fold difference from the smallest to largest within-person variance for the individual mean scores.
